# M. Recamier’s Aloetico-Febrifugic Elixir

**Published:** 1850-03

**Authors:** 


### MATERIA MEDICA AND THERAPEUTICS.

*M. Recamier's Aloetico-Febrifugic Elixir.*—The obstinacy of certain intermittent fevers and neuralgic affections is familiarly known, and when it is sought to contend against this by largely increasing the dose of quinine, very mischievous results sometimes follow, especially in persons of constipated habit, who then often suffer from long-continued disturbance of the sight and hearing. The following formula devised by M. Recamier, has, in his hands, and in those of various other practitioners, been found of great utility in these cases:

| | |
|---|---|
| Powdered socotrine aloes, - - | 6 parts |
| Picked myrrh, - - - | 6 " |
| Rum, - - - - - - - | 150 " |
| Alcohol, - - - - - | 20 " |

Macerate for twenty-four hours, and add to the filtered liquor—

| | |
|---|---|
| Sulph. quinine (previously acidulated) | 6 parts |
| Sydenham's laudanum - - - | 2 " |

The dose is a teaspoonful for children, and a tablespoonful for adults.

The patient should keep himself warm in bed, after each dose, and should abstain from drink for at least two hours. In rheumatic affections, additional advantage is derived by adding four parts of powdered colchicum bulbs.—*Brit. and For. Med. Chir. Rev. from Jour. de Phar.*

